# Work-Related Maternal Risk Factors and the Risk of Pregnancy Induced Hypertension and Preeclampsia during Pregnancy. The Generation R Study

**DOI:** 10.1371/journal.pone.0039263

**Published:** 2012-06-15

**Authors:** Jaap Jan Nugteren, Claudia A. Snijder, Albert Hofman, Vincent W. V. Jaddoe, Eric A. P. Steegers, Alex Burdorf

**Affiliations:** 1 The Generation R Study Group, Erasmus MC, Rotterdam, The Netherlands; 2 Department of Public Health, Erasmus MC, Rotterdam, The Netherlands; 3 Department of Epidemiology, Erasmus MC, Rotterdam, The Netherlands; 4 Department of Paediatrics, Erasmus MC, Rotterdam, The Netherlands; 5 Department of Obstetrics and Gynaecology, Erasmus MC, Rotterdam, The Netherlands; Hospital Clinic, University of Barcelona, Spain

## Abstract

**Objective:**

To study the associations between physically demanding work and occupational exposure to chemicals and hypertensive disorders during pregnancy within a large birth cohort study, the Generation R Study.

**Methods:**

Associations between occupational characteristics and hypertensive disorders during pregnancy were studied in 4465 pregnant woman participating in a population-based prospective cohort study from early pregnancy onwards in the Netherlands (2002–2006). Mothers who filled out a questionnaire during mid-pregnancy (response 77% of enrolment), were included if they conducted paid employment, had a spontaneously conceived singleton live born pregnancy, and did not suffer from pre-existing hypertension (n = 4465). Questions on physical demanding work were obtained from the Dutch Musculoskeletal Questionnaire and concerned questions on manually handling loads of 25 kg or more, long periods of standing or walking, night shifts, and working hours. To assess occupational exposure to chemicals, job titles and task descriptions were linked to a job-exposure-matrix (JEM), an expert judgment on exposure to chemicals at the workplace. Information on hypertensive disorders during pregnancy was obtained from medical records.

**Results:**

We observed no consistent associations between any of the work related risk factors, such as long periods of standing or walking, heavy lifting, night shifts, and working hours, nor exposure to chemicals with hypertensive disorders during pregnancy.

**Conclusion:**

This prospective birth cohort study suggests that there is no association of hypertensive disorders during pregnancy with physically demanding work or exposure to chemicals. However, the low prevalence of PIH and PE, combined with the low prevalence of occupational risk factors limit the power for inference and larger studies are needed to corroborate or refute these findings.

## Introduction

Hypertensive disorders during pregnancy are among the leading causes of maternal and neonatal morbidity worldwide, and include pregnancy induced hypertension (PIH) and preeclampsia [1], [2]. PIH and preeclampsia complicate about 7% of all pregnancies [3] and severe preeclampsia is a major cause of severe maternal morbidity (e.g. stroke and liver rupture) and adverse perinatal outcomes, such as prematurity and intrauterine growth restriction [4]. Risk factors for PIH and preeclampsia include family or obstetric history of preeclampsia, first pregnancy, obesity, higher maternal age, pre-existing diabetes, renal disease, hypertension, and chronic autoimmune disease [5]–[9]. Evidence for the influence of environmental and occupational factors is contradictory to a few studies that have suggested that these factors may play a role in the etiology of hypertensive disorders during pregnancy [10]. However, the underlying mechanisms for occupational risk factors, such as physically demanding work and exposure to chemicals, are unclear.

Physically demanding work, such as prolonged standing and frequent lifting, may increase catecholamine levels [11]–[13] which may affect constriction/dilatation of blood vessels [14]. High levels of catecholemines have been demonstrated in patients suffering from preeclampsia [15]. Furthermore, increased catecholamine levels are hypothesized to decrease uterine blood flow and may therefore influence early placentation [12]. Contradictory findings have been reported on physically demanding work and occurrence of PIH or preeclampsia. Mozurkewich et al. showed in a meta-analysis, based on 4 studies, that physically demanding work was significantly associated with PIH and preeclampsia (OR 1.60, 95%CI 1.30–1.96) [16]. A more recent and larger review by Bonzini et al., based on 8 studies, concluded that for preeclampsia and PIH, although several positive findings were reported, the evidence base was too limited to allow firm conclusions. This second review excluded less articles than Mozurkewich et al., and included five more years of research, covering almost twice the number of articles. No meta-analysis could be performed, due to the large heterogeneity in exposure definitions, and the available evidence was not sufficient to justify mandatory restrictions on any of the occupational activities during pregnancy [17]. This latter review included some new studies that showed modest to no effect of several aspects of physically demanding work, such as working hours, standing, lifting, physical activity, and shift work on PIH and preeclampsia [18]–[23]. However, a recent study by Haelterman et al., which was not included in either review, showed that prolonged standing was associated with an increased risk of preeclampsia [24].

Occupational exposure to chemicals in relation to hypertensive disorders during pregnancy has been rarely studied. Some studies on maternal exposure to chemicals have suggested that organic solvents [25] and pesticides [26] may increase the risk of hypertensive disorders. Based on these previous studies, we hypothesized that occupational risk factors, such as physically demanding work and exposure to chemicals, may influence the occurrence of PIH or preeclampsia. Since studies on occupational risk factors showed conflicting results, it is unclear how working pregnant women should be managed. Further studies are needed to elucidate the role of occupational risk factors in the pathogenesis of PIH and preeclampsia, such that preventive measures, if needed, can be taken.

The aim of this study was to assess, in a population-based prospective cohort study, the associations between physically demanding work and exposure to chemicals with hypertensive disorders during pregnancy.

## Materials and Methods

### Design and study population

**Figure 1:**
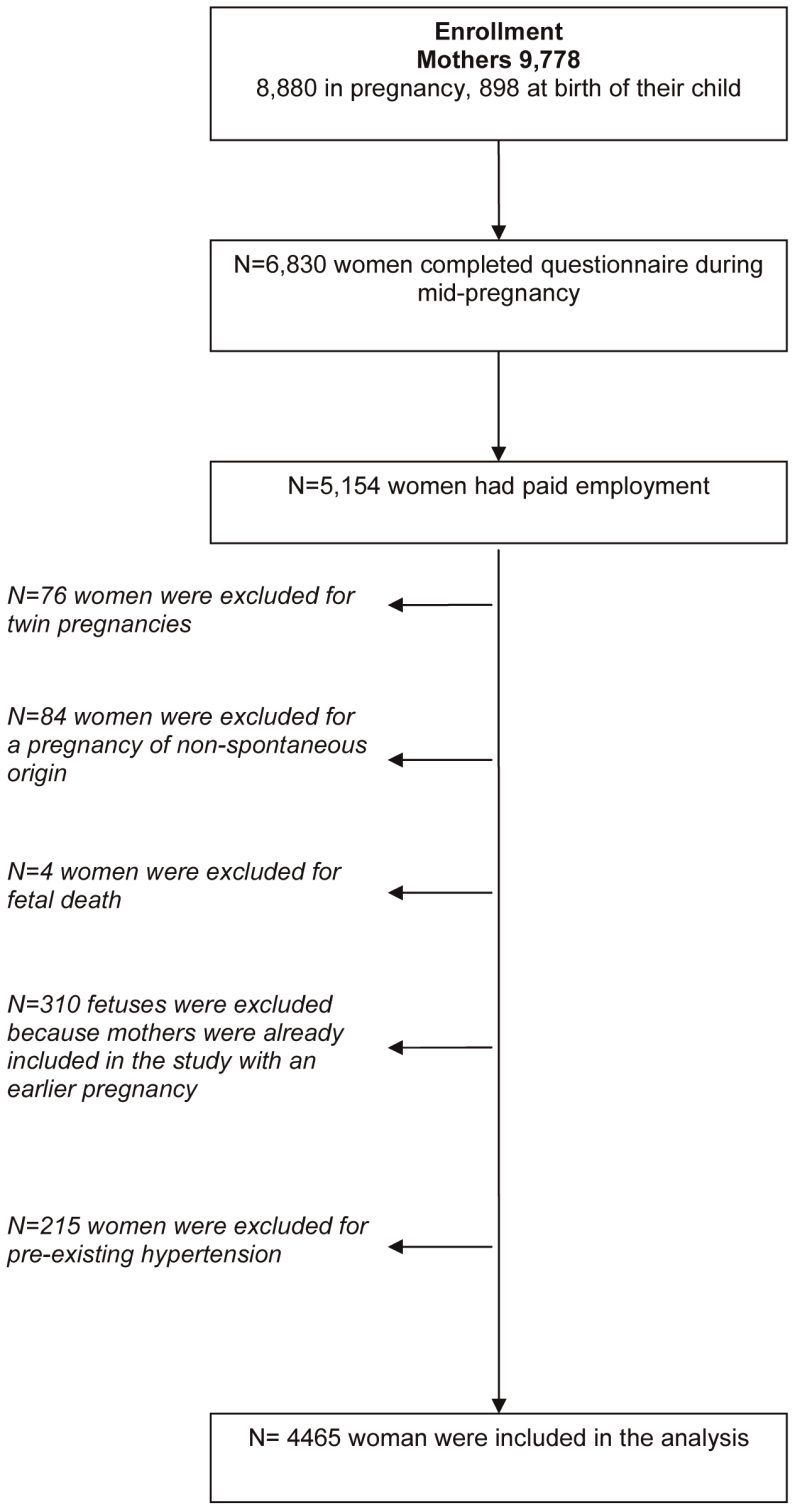
Flowchart of the study population.

The Generation R Study is a population-based prospective cohort study on growth, development, and health from early fetal life until young adulthood in Rotterdam, The Netherlands. The study design has been described in detail previously [27]. Briefly, all pregnant women who had an expected delivery date between April 2002 and January 2006 and living in the study area of Rotterdam were invited to participate. In total, 9,778 women (response 61%) were enrolled in the study of which 8,880 women during pregnancy and another 898 at the birth of their child. The information required for this study was collected in the questionnaire completed during mid-pregnancy (around approximately 30 weeks of gestation) by 6,830 women (77% of enrolment) and information on pregnancy complications was obtained from medical records. For this study we selected women who were prenatally enrolled, with paid employment before or during pregnancy, with no history of pre-existing hypertension (blood pressure ≥140/90 mmHg before 20 weeks' gestation) [4] and with a spontaneously conceived singleton liveborn pregnancy (n = 4465). Spontaneously conceived refers to pregnancies achieved without assisted reproductive techniques, such as ovulation induction or in vitro fertilisation. For each mother, we included the first pregnancy within the Generation R cohort in our study, since some women participated with more than one child in the study. The flowchart of the study population is depicted in Figure 1. The study was approved by the Medical Ethics Committee at Erasmus University Medical Centre Rotterdam, The Netherlands (MEC 198.782/2001/31). Written consent was obtained from all participants.

### Hypertensive disorders during pregnancy

Information on pregnancy complications was obtained from medical records. Women who delivered in hospital and who had chronic hypertension or were reported to have experienced PIH (>140/90 mm Hg) or hypertension related complications (preeclampsia, proteinuria, eclampsia, and/or HELLP syndrome), were selected from hospital registries. Their individual medical records were subsequently studied by qualified medical doctors, who defined pregnancy induced hypertension, preeclampsia and eclampsia according to the criteria of the International Society for the Study of Hypertension in Pregnancy (ISSHP) and according to those of the College of Obstetricians and Gynaecologists (ACOG). Blood pressure measurements were performed during pregnancy in early, mid and late pregnancy until gestational week 32–34. Details of these procedures have been described elsewhere [28]. Briefly, the following criteria were used to identify woman with PIH: development of systolic blood pressure ≥140 mm Hg and/or diastolic blood pressure ≥90 mm Hg after 20 weeks of gestation in previously normotensive woman. These criteria plus the presence of proteinuria (defined as two or more dipstick readings of 2+ or greater, one catheter sample reading of 1+ or greater, or a 24-hour urine collection containing at least 300 mg of protein) were used to identify woman with preeclampsia [29].

### Occupation and working conditions

The mid-pregnancy questionnaire contained questions about work status, occupation, and working conditions and focussed on the periconception and pregnancy period. Work status, based on a single question on the current economic status with seven categories (paid labour, self-employed, unemployed, disabled, homemaker, student, or other), was used to select women with paid employment, consisting of women within the first two categories. This question was followed by questions whether the mother had worked before conception in this current occupation, and the starting and (optional) stop date of this current occupation. We selected women who started working before conception and women who started working somewhere during the first trimester of pregnancy. Further questions on job title, type of business, name of employer, and activities in the job were used to classify jobs into the Dutch Classification of Occupations [30] and subsequently link these codes to a Job-Exposure-Matrix (JEM) for chemical exposure. This new JEM was developed according to a general strategy, comprising a literature search to identify chemicals, information gathering on occupations at risk and literature on occupational settings in which the selected chemicals were encountered and exposure measurements were performed. This reference material served as a starting point for the expert assessment. Three experts were asked to estimate exposures based on their knowledge of tasks and working environment in various occupations. Finally, exposure probability scores were added based on the judgement of three experts. For various chemicals, subjects experience a certain level of exposure through diet, environment or widely used consumer products. The JEM exposure score refers to the probability of occupational exposure, which is assumed to exceed the background level in the general population. The exposure probability scores were assigned by means of consensus discussions in which the original scores were taken into account where possible, but no prior individual assessments were performed. The JEM comprises 10 categories of chemicals, namely polycyclic aromatic hydrocarbons (PAHs), polychlorinated organic compounds, pesticides, phthalates, organic solvents, bisphenol A, alkylphenolic compounds, flame retardants, metals and miscellaneous agents [31]. For 353 job titles, probability scores were classified into three levels: ‘unlikely’(0), ‘possible’(1), and ‘probable’(2). For this study we collate the last two categories into one category indicating possible exposure to chemicals. The category “any chemicals” combines all women exposed to one of the groups of chemicals defined in the JEM. Different country specific JEMs have been used in various studies, and the JEM is a valuable tool for exposure assessment in epidemiological studies on the health risks of chemical exposure [31]–[35]. The questions on physically demanding work were based on the Dutch Musculoskeletal Questionnaire [36] and concerned questions on manually handling loads of 25 kg or more, long periods of standing, long periods of walking, long periods of driving, night shifts, and working hours. These questions were part of the mid-pregnancy questionnaire distributed around approximately 30 weeks of gestation. A four-point scale was used with ratings ‘seldom or never’, ‘occasionally’, ‘often’, and ‘very often’ during a regular workday [36]. We reclassified long periods of standing and walking into three categories, namely ‘seldom or never’, ‘occasionally’, and ‘often/very often’. We reclassified long periods of driving, manual handling of load of 25 kg or more and night shifts into two categories, namely ‘seldom or never’ and ‘occasionally/often/very often’. The number of weekly working hours of the mothers with paid employment was assessed by means of an open question, ‘How many hours per week do you work?’. Working hours were categorised into ‘1–24’, ‘25–39’, and ‘40 or more hours a week’ [37].

### Potential confounders

The following variables were considered as possible confounders in the association between physically demanding work, exposure to chemicals and hypertensive disorders during pregnancy: maternal age, pre-pregnancy weight, height, educational level, ethnicity, parity, smoking, alcohol use, and folic acid supplement use. Information about maternal age, educational level, ethnicity, parity, and folic acid supplement use was obtained by questionnaire at enrolment in the study. Maternal smoking habits and alcohol use were assessed in three prenatal questionnaires in each trimester and classified into three categories, namely no smoking or alcohol use, smoking or alcohol use until pregnancy was known, and smoking or alcohol use during pregnancy [38]. Maternal height was measured at intake in the study. Body mass index (BMI) was calculated as weight divided by squared height.

### Statistical analysis

We used bivariate and multivariate logistic regression analyses to study the association between maternal characteristics, occupational risk factors and hypertensive disorders during pregnancy. We reclassified age, a continuous variable, into four categories for ease of interpretation. Individual characteristics significantly associated with PIH or preeclampsia were considered for the multivariate analyses. The final model consisted of the following confounders: maternal age, educational level (both included by default), ethnicity, parity, and BMI. Two sensitivity analyses were carried out, first we stratified the analyses for Dutch versus non-Dutch women, secondly we assessed whether women who quitted working before 34 weeks of gestation because of pregnancy complaints had a higher risk of PIH or preeclampsia. This information on the gestational week women stopped working was available for 3537 women (68.6%). Missing values in confounders were handled by multiple imputations (fully conditional specification, Markov Chain Monte Carlo method) by generating 5 independent datasets for all analyses, using SPSS version 17.0 for windows. Variables included in the imputation procedure (these variables were both imputed and used as predictors of missing data) were: maternal age, educational level, ethnicity, parity, pre-pregnancy weight, height at intake, smoking during pregnancy, alcohol use during pregnancy, folic acid supplement use, fetal gender, and gestational age at birth. Table 1 presents the proportion of missing values for each variable that was imputed. All multivariate analyses were performed with the multiple imputation datasets, and pooled estimates were calculated across these five independent datasets. The maximal allowed threshold for imputations was set on a maximum of missing values of 30% [39]. All logistic regression analyses were performed using Statistical Package of Social Sciences version 17.0 for Windows (SPSS Inc, Chicago, IL, USA).

**Table 1:** Baseline characteristics of pregnant women participating in a birth cohort study, the Generation R Study (n = 4465).

Maternal characteristics	Subcategory	Results
Age at intake (years)		31.09 (4.5)
Weight before pregnancy (kg)		69.35 (12.44)
Height measured at intake (cm)		168.78 (7.14)
Educational level	Low	611 (13.7%)
	Mid-low	1273 (28.5%)
	Mid-high	1076 (24.1%)
	High	1365 (30.6%)
	Missing	140 (3.1%)
Ethnicity	Netherlands	2845 (63.7%)
	Surinam and Dutch Antilles	360 (8.1%)
	Morocco and Turkey	322 (7.2%)
	Other	847 (19.0%)
	Missing	91 (2.0%)
Parity	Nulliparous	2826 (63.3%)
	Multiparous	1520 (34.0%)
	Missing	119 (2.7%)
Body Mass Index (BMI)	<25 kg/m^2^	2724 (61.0%)
	25–30 kg/m^2^	1016 (22.8%)
	>30 kg/m^2^	380 (8.5%)
	Missing	345 (7.7%)
Smoking	No	2899 (64.9%)
	Yes, until pregnancy was known	332 (7.4%)
	Yes, during pregnancy	519 (13.8%)
	Missing	715 (16.0%)
Alcohol	No	1459 (32.7%)
	Yes, until pregnancy was known	559 (12.5%)
	Yes, during pregnancy	1757 (39.4%)
	Missing	690 (15.5%)
Folic acid use	No	555 (12.4%)
	Yes, post conception start	1097 (24.6%)
	Yes, preconception start	1662 (37.2%)
	Missing	1151 (25.8%)
Hypertensive disorders during pregnancy	Preeclampsia	60 (1.3%)
	Pregnancy induced hypertension	79 (1.8%)

Values are means (SD) for normal distributed continuous variables or medians (minimum–maximum) for skewed distributed continuous variables, and absolute numbers (percentages) for categorical variables.

## Results

The characteristics of the study population are shown in Table 1. Age at enrolment ranged from 17.0 to 46.0 years with a mean age of 31.1 years. The largest ethnic group was from Dutch origin (63.7%), and Surinamese and Dutch Antillean women (8.1%), and Turkish and Moroccan women (7.2%) were less represented. The prevalence of pregnancy-induced-hypertension (PIH) and preeclampsia in our study population was 1.8% (79 cases) and 1.3% (60 cases), respectively. The occupational characteristics are presented in Table 2.

**Table 2:** Occupational characteristics of pregnant women participating in a birth cohort study, the Generation R Study (n = 4465).

Occupational characteristics	Subcategory	Results
Long periods of standing	No	2329 (52.2%)
	Occasionally	881 (19.7%)
	Often/very often	840 (18.8%)
	Missing	415 (9.3%)
Long periods of walking	No	2036 (45.6%)
	Occasionally	1399 (31.3%)
	Often/very often	634 (14.2%)
	Missing	396 (8.9%)
Long period of driving	No	3499 (78.4%)
	Occasionally/often/very often	572 (12.8%)
	Missing	394 (8.8%)
Lifting or carrying weights >25 kg	No	3815 (85.4%)
	Occasionally/often/very often	267 (6.0%)
	Missing	383 (8.6%)
Night shift (each month)	No	3892 (87.2%)
	Occasionally/often/very often	188 (4.2%)
	Missing	385 (8.6%)
Working hours	<25 hours per week	1163 (26.0%)
	25–39 hours per week	2112 (47.3%)
	>40 hours per week	1040 (23.3%)
	Missing	150 (3.4%)
Exposure to chemicals (JEM)	PAH	55 (1.2%)
	Pesticides	22 (0.5%)
	Phthalates	65 (1.5%)
	Organic solvents	213 (4.8%)
	Alkylphenolic compounds	150 (3.4%)
	Metals	51 (1.1%)
	Any chemicals	297 (6.7%)

Values are absolute numbers (percentages).

The bivariate analysis in Table 3 shows associations between individual characteristics and hypertensive disorders during pregnancy. Multiparous women were at lower risk for both PIH and preeclampsia. Compared to Dutch mothers, women from other ethnic minorities showed a lower risk on PIH (OR 0.47; 95%CI 0.23–0.94). For preeclampsia, we observed that Surinamese and Dutch Antillean women showed a significantly higher risk of preeclampsia (OR 2.23; 95%CI 1.08–4.57). Overweight and obese mothers had increased risks of PIH and preeclampsia. Smoking and alcohol consumption were not associated with PIH or preeclampsia.

**Table 3:** Associations in a birth cohort study among pregnant women on maternal individual characteristics and hypertensive disorders during pregnancy.

Maternal characteristics	Subcategory	PIH	Preeclampsia
		OR (95% CI)	OR (95% CI)
Age before intake	<25 years	1.00	1.00
	25–29 years	2.07 (0.79–5.46)	2.39 (0.82–6.95)
	30–35 years	1.70 (0.66–4.37)	1.67 (0.58–4.81)
	>35 years	2.19 (0.80–6.01)	1.20 (0.35–4.11)
Educational level	Low	0.82 (0.39–1.72)	1.91 (0.90–4.07)
	Mid-low	1.05 (0.61–1.82)	1.77 (0.92–3.40)
	Mid-high	0.82 (0.44–1.51)	0.53 (0.19–1.44)
	High	1.00	1.00
Ethnicity	Netherlands	1.00	1.00
	Surinam and Dutch Antilles	0.71 (0.31–1.67)	2.23 (1.08–4.57)*
	Morocco and Turkey	0.26 (0.06–1.05)	1.29 (0.50–3.34)
	Other	0.47 (0.23–0.94)*	1.10 (0.55–2.20)
Parity	Nulliparous	1.00	1.00
	Multiparous	0.55 (0.33–0.94)*	0.21 (0.09–0.49)**
Body Mass Index (BMI)	<25 kg/m^2^	1.00	1.00
	25–30 kg/m^2^	2.86 (1.66–4.93)**	2.19 (1.26–3.80)*
	>30 kg/m^2^	7.96 (4.57–13.88)**	2.20 (1.00–4.84)
Smoking	No	1.00	1.00
	Yes, until pregnancy was known	0.92 (0.39–2.17)	0.37 (0.09–1.54)
	Yes, during pregnancy	0.99 (0.48–2.01)	0.81 (0.36–1.80)
Alcohol	No	1.00	1.00
	Yes, until pregnancy was known	0.98 (0.50–1.92)	0.99 (0.45–2.16)
	Yes, during pregnancy	0.85 (0.51–1.42)	0.89 (0.51–1.57)
Folic acid use	No	1.00	1.00
	Yes, post conception start	1.61 (0.74–3.51)	1.16 (0.52–2.55)
	Yes, preconception start	1.44 (0.69–3.01)	1.00 (0.48–2.11)

* p<0.05.

** p<0.01.

Table 4 shows the bivariate and multivariate logistic regression analyses between occupational risk factors and hypertensive disorders during pregnancy. There were no consistent associations between physically demanding work and chemical exposure with hypertensive disorders during pregnancy. For almost all risk factors related to physically demanding work we could not find a clear exposure-response relation, and women “often” exposed to a certain occupational risk factor were not consistently at higher risk for hypertensive disorders during pregnancy compared to women who were “occasionally” exposed. When we restricted the analysis to primigravidous women (63% of the study population), the effect estimates remained very similar to the presented effect estimates in Table 4 (data not shown). Furthermore, there were no differences in effect estimates between Dutch and non-Dutch women. Women quitting their job before 34 weeks of gestation were significantly at higher risk of PIH (OR 1.81; 95%CI 1.04–3.14) and at higher risk of preeclampsia (OR 1.92; 95%CI 0.96–3.84), although not statistically significant (data not shown).

**Table 4:** Associations in a birth cohort study among pregnant women on physically demanding work, chemical exposure and hypertensive disorders during pregnancy.

		PIH		Preeclampsia	
Occupational characteristics	Subcategory	OR (95% CI)	aOR (95% CI)	OR (95% CI)	aOR (95% CI)
Long periods of standing	No	1.00	1.00	1.00	1.00
	Occasionally	1.02 (0.58–1.80)	1.05 (0.59–1.88)	1.12 (0.60–2.11)	1.01 (0.52–1.93)
	Often/very often	1.00 (0.56–1.78)	1.16 (0.62–2.15)	1.00 (0.51–1.94)	0.87 (0.43–1.78)
Long periods of walking	No	1.00	1.00	1.00	1.00
	Occasionally	1.55 (0.95–2.55)	1.68 (1.00–2.81)*	0.82 (0.46–1.47)	0.74 (0.41–1.35)
	Often/very often	1.45 (0.77–2.74)	1.74 (0.87–3.47)	1.00 (0.49–2.05)	0.77 (0.37–1.67)
Long periods of driving (>4 hours)	No	1.00	1.00	1.00	1.00
	Occasionally/often/very often	0.75 (0.42–1.34)	1.30 (0.87–1.47)	0.93 (0.45–1.89)	0.77 (0.37–1.60)
Lifting or carrying weights >25 kg	No	1.00	1.00	1.00	1.00
	Occasionally/often/very often	0.84 (0.36–1.95)	0.92 (0.39–2.18)	0.98 (0.35–2.72)	1.07 (0.38–3.01)
Night shifts (each month)	No	1.00	1.00	1.00	1.00
	Occasionally/often/very often	0.57 (0.24–1.32)	0.59 (0.25–1.42)	0.89 (0.28–2.88)	0.86 (0.26–2.80)
Working hours	<25 hours per week	1.00	1.00	1.00	1.00
	25–40 hours per week	0.91 (0.53–1.54)	0.67 (0.38–1.20)	1.14 (0.57–2.26)	0.81 (0.40–1.66)
	>40 hours per week	0.71 (0.32–1.38)	0.43 (0.20–0.90)*	1.74 (0.85–3.59)	1.04 (0.48–2.26)
Exposure to chemicals (JEM)	PAH	2.99 (0.91–9.77)	2.64 (0.74–9.35)	1.28 (0.17–9.43)	0.89 (0.12–6.75)
	Pesticides	.	.	3.14 (0.42–23.73)	3.15 (0.38–25.94)
	Phthalates	.	.	1.05 (0.14–7.72)	0.82 (0.11–6.16)
	Organic solvents	0.72 (0.22–2.29)	0.94 (0.29–3.09)	0.96 (0.30–3.08)	0.92 (0.28–3.04)
	Alkylphenolic compounds	1.04 (0.32–3.34)	1.56 (0.46–5.29)	0.91 (0.22–3.75)	0.81 (0.19–3.45)
	Metals	.	.	2.72 (0.65–11.43)	2.21 (0.50–9.67)
	Any chemicals	1.05 (0.45–2.44)	1.22 (0.51–2.94)	1.17 (0.46–2.93)	1.04 (0.40–2.68)

* p-value <0.05.

Effect estimates were adjusted for maternal age, educational level, parity, ethnicity and BMI.

## Discussion

In this large population-based prospective birth cohort study we were not able to find consistent associations between physically demanding work and exposure to chemicals with hypertensive disorders during pregnancy. These results suggest that there is no effect of occupational risk factors on the occurrence of hypertensive disorders during pregnancy. The main limitation of this study is the limited number of women with PIH or PE and the low prevalence of exposure to chemicals.

The findings in our study corroborates with the conclusions from a recent review that the available evidence on the presence of an association between physically demanding work and PIH or preeclampsia was not sufficient to propose restrictions in activities during pregnancy [17]. However, another review reported a clear association between physically demanding work during pregnancy and preeclampsia [16]. We hypothesized that the contradictory findings in the scientific literature may be partly due to heterogeneity in the definition of PIH and preeclampsia across studies, and also in the definitions of physically demanding work, which makes comparisons difficult. In our study, we used strict criteria to assess hypertensive complications during pregnancy. Medical records were checked and the diagnosis was made by qualified medical doctors. The low prevalence of these disorders in our study population can be explained by the strict criteria for diagnosis. Furthermore, blood pressure measurements in our study were performed until gestational week 32–34. Thereafter, medical records were checked for the occurrence of PIH and preeclampsia, this might have led to a lower incidence of PIH, since this disease may have no clear pattern of symptoms, and often, hospital admission is not required. Another explanation for the low prevalence may be the selection of women with paid employment, since these women generally have better pregnancy outcomes than women without paid employment [32], [37]. In our analyses, we choose women with paid employment, to avoid “health worker bias” [37]. Furthermore, women with pregnancy complications may quit their job earlier during pregnancy than healthy women, and technically these women would be on sick leave. The sensitivity analyses on women who reported stopping working before 34 weeks because of pregnancy complaints showed that these women were at higher risk of PIH and PE. However, quitting before 34 weeks of gestation was not associated with physically demanding work nor exposure to chemicals and, thus, will not have influenced the reported associations. Since women from ethnic minorities may also have higher risks of adverse pregnancy outcomes, we carried out stratified analyses, however, effect estimates were comparable, indicating no differences.

For occupational exposure to chemicals and hypertensive disorders during pregnancy, the evidence is scarce and contradictory. Irwin et al. found no relation between occupational exposure to chemicals and hypertensive disorders during pregnancy [18], whereas Eskenazi and Saldana reported associations between solvents and pesticides with PIH and preeclampsia [25], [26]. In our study, exposure to pesticides showed an increased risk of preeclampsia (OR 3.15; 95%CI 0.38–25.94). However, this was not statistically significant, probably due to the low number of women exposed to pesticides (n = 23). We must conclude that the prevalence of occupational exposure to chemicals in the general population is very low, and, thus, the proportion of PIH and preeclampsia attributable to occupational exposure will be low.

One of the suggested mechanism through which physically demanding work could lead to hypertensive disorders during pregnancy is an increased uteroplacental vascular resistance which follows physical exertion [40]. Physically demanding work may cause an increase in catecholamine levels, which may lead to a decreased uterine blood flow and therefore may induce PIH and preeclampsia [12]. It has also been suggested that part of the excess catecholamine release is due to an overactive sympathetic nervous system [41]. For exposure to chemicals, the underlying mechanisms are largely unclear.

Exposure assessment is an important issue in this study. For assessment of maternal exposure to chemicals we used a recently updated Job-Exposure-Matrix (JEM) [31]–[35]. This approach assured that exposures status was blinded to participants and researchers, both aspects which avoid information bias. The characterization of exposure in the JEM must be interpreted as exposure probabilities. However, if misclassification occurred, this is most likely non-differential misclassification, leading to underestimation of the effect estimates. A major drawback of JEMs is that they do not account for variability in tasks and working environments within job titles. Furthermore, the JEM does not contain specific chemicals, but only contains broad groups of chemicals, and the mechanisms of action can vary between specific chemicals in a group. However, from the task description, it may become clear that some subjects within a specific job title, for example subjects who have odd jobs around a farm (feeding animals) are less likely to be exposed to pesticides. Background exposure to various chemicals through diet and environment may occur. However, it is unlikely that background exposure is associated with occupational exposure, thus, background exposure will not confound the relation between occupational chemical exposure and hypertensive disorders during pregnancy. Furthermore, it is expected that levels of exposure to chemicals within occupations are generally much higher than general exposure through diet and environment. Since we did not assess background exposure, it may have contributed to unexplained variance in the outcome hypertensive disorders during pregnancy. In our study we classified physically demanding work in three or two relevant levels of exposure, however, this approach does not quantify the exposure into hours of physically demanding work performed per day, and therefore is at best a semi-quantitative measure. Furthermore, this study did not take into account other sources of physically demanding activities outside employment, such as exercise, housework, and volunteer work. However, it is unlikely that these activities are strongly related to physically demanding work risk factors, but they may lead, in some extent, to residual confounding.

In order to assess whether there was any overlap between the occupational risk factors in this study, we calculated kappa values for all exposure categories. Kappa values ranged from 0.00–0.18, indicating that there was almost no overlap between physically demanding work and exposure to chemicals in the workplace.

The strength of this study is the population-based approach with recruitment during the prenatal period and the availability of a large number of potential risk factors. Within the Generation R cohort, Bakker et al. showed that smoking during the first trimester is associated with maternal cardiovascular adaptations during pregnancy [42]. Gaillard et al. showed that there is a strong relation between obesity and PIH and preeclampsia [43]. Thus, in our analysis we could adjust for these well-established risk factors. A limitation of this study is the selective participation whereby mothers from ethnic minorities, those with lower socio-economic status, and mothers or children with medical complications, were less represented in the study population than expected in the population of Rotterdam [44]. This non-response will lead to biased effect estimates if the association between physically demanding work, chemical exposure and hypertensive disorders during pregnancy differs between participants and non-responders. However, this seems unlikely since biased estimates in large cohort studies mainly arise from loss to follow up rather than from non-response at baseline [45]. Selective participation may have influenced the prevalence of exposure to physically demanding work and chemicals, but bias is unlikely since physically demanding work and exposure to chemicals was assessed independently from and prior to the hypertensive disorders during pregnancy. Although we were able to control for a large number of potential confounders, residual confounding cannot be ruled out completely. Recall bias in this study is unlikely, since the information obtained was not biased by the outcome since the questionnaire was completed in mid-pregnancy. In this study we used multiple imputation for missing values in covariates. This reduces bias due to non-random missing in the covariates.

In summary, this large population-based birth cohort study suggests that physically demanding work and exposure to chemicals did not influence the occurrence of hypertensive disorders during pregnancy. However, the very low prevalence of PIH and PE in our study, combined with the low prevalence of occupational risk factor, may have resulted in too little discriminatory power to detect such associations.
